# Estimation of the maximum utilization area including home range and peripheral sites

**DOI:** 10.1002/ece3.8893

**Published:** 2022-05-07

**Authors:** Kana Terayama, Hiroshi Ebihara, Hironori Seino, Motomi Genkai‐Kato

**Affiliations:** ^1^ 12888 Graduate School of Kuroshio Science Kochi University Kochi Japan; ^2^ Wildlife Management Office Kobe Japan

**Keywords:** asymptote, maximum utilization area, minimum convex polygon, periphery, wildlife

## Abstract

There is increasing evidence that occasional utilization area (peripheral sites), in addition to typical utilization area (home range), is important for wildlife conservation and management. Here we estimated the maximum utilization area (MUA), including both typical and occasional utilization areas, based on asymptotic curves of utilization area plotted against sample size. In previous studies, these curves have conventionally been plots of cumulative utilization area versus sample size, but this cumulative method is sensitive to stochastic effects. We propose a new method based on simulation studies where outcomes of replicated simulations are averaged to reduce stochastic effects. In this averaged method, possible combinations of sample size with the same number of location data replicated from a dataset were averaged and applied to the curves of utilization area. The cumulative method resulted in a large variation of MUA estimates, depending on the start date as well as total sample size of the dataset. In the averaged method, MUA estimates were robust against changes in the start date and total sample size. The large variation of MUA estimates arose because location data on any day including the start date are affected by unpredictable effects associated with animal activity and environmental conditions. In the averaged method, replicates of sample size resulted in a reduction of temporal stochasticity, suggesting that the method stably provides reliable estimates for MUA.

## Graphical abstract

Because there is increasing evidence that occasional utilization area (peripheral sites), in addition to typical utilization area (home range), is important for wildlife conservation and management, we estimated the maximum utilization area (MUA), including both typical and occasional utilization areas. Here we propose a new method to estimate MUA, following simulation studies where outcomes of replicated simulations are averaged to reduce stochastic effects. With our new method, replicates of sample size resulted in a reduction of temporal stochasticity, suggesting that our method stably provides reliable estimates for MUA.

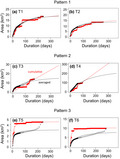

## 1 INTRODUCTION

Peripheral sites outside of home ranges can be important in theoretical and applied ecology such as wildlife conservation and management for three reasons. First, encounters between animals often occur in peripheral sites which are the spatial area of overlap between the home ranges of two or more individuals. Encounters are closely related to the life of animals through intra‐ and inter‐specific interactions such as predator–prey relationships and mating (Long et al., 2015; Martinez‐Garcia et al., 2020). Second, incidents related to human–wildlife conflict have often been reported at peripheral sites. In South Africa, domestic sheep are often attacked by black‐backed jackals outside the estimated home ranges of the carnivores (Kamler et al., 2019). In California, USA, gray foxes have higher mortality risk outside or at the periphery of their home ranges due to predation by coyotes and bobcats (Farias et al., 2005). Third, peripheral sites often provide valuable locations to conserve endangered species (Channell & Lomolino, 2000). The International Union for the Conservation of Nature (IUCN) recognized this importance of peripheral sites when it developed the concept of "extent of occurrence (EOO)," which is the area contained within the boundary encompassing all the known, inferred or projected sites of present occurrence of a taxon (IUCN, 2001).

There are a variety of approaches to estimate animal space use based on location data. Among these approaches, minimum convex polygon (MCP) and kernel density estimation (KDE) have been the most commonly used (Laver & Kelly, 2008). In the MCP approach, the estimated area encompasses all location data including occasional locations (i.e., peripheral sites) beyond the main area of activity. The KDE approach is based on the density of locations and provides an estimation of the main area of activity. For estimation of the maximum utilization area (MUA) including peripheral sites, MCP is considered the superior estimator to KDE (Keuling et al., 2008). In fact, the MCP approach is strongly recommended for estimation of EOO (IUCN Standards & Petitions Committee, 2019).

Recent advances in animal tracking technology such as GPS have allowed researchers to collect location data of animals accurately (Tomkiewicz et al., 2010). However, GPS devices are inevitably subject to the trade‐off between observation period and frequency of data collection because of their limited battery life (Brown et al., 2012). This means that the total number of location data (sample size) is limited depending on the battery life. Because estimates of utilization area should reach an asymptote with an adequate sample size (Laver & Kelly, 2008; McLoughlin & Ferguson, 2000; Odum & Kuenzler, 1955), assessment of an adequate sample size has been conducted in a variety of animal species. Previous studies on sample‐size assessment with observed data usually plotted the utilization area against sample size in a cumulative manner (Barros & Motte‐Junior, 2014; Bertassoni et al., 2020; Majumder et al., 2012; Smith & Mathieson, 2007). These studies showed that the curves of utilization area often do not reach an asymptote, presumably due to unpredictable effects associated with animal activity and environmental conditions. For example, a dataset with a limited observation period starting from relatively inactive days (e.g., due to predator avoidance or rainy days) is likely to result in a curve that does not reach an asymptote when the curve is plotted in a cumulative manner. In simulation studies, researchers usually perform a number of replicates and simulated outcomes are averaged to reduce stochastic effects (Beckoff & Mech, 1984; Seaman & Powel, 1996). Following these simulation approaches, we obtained subsamples from the original dataset as replicates of a series of sample sizes to reduce the effect of daily variation in utilization area. Using these subsamples, the utilization area calculated by the 100%‐MCP approach was averaged within the same sample size and it was plotted against a series of sample sizes. The MUA estimated using the subsamples (averaged method) was compared with MUA estimated by the cumulative method.

## 2 MATERIALS AND METHODS

### 2.1 Study animal and sites

The Japanese macaque (*Macaca fuscata*) was used as our model species. We focused on six troops (T1–T6) of this species, located on Honshu Island (main island of Japan) and Shikoku Island (Table 1). These troops inhabit Satoyama, a Japanese traditional socio‐ecological landscape including paddy fields, secondary and coniferous forests, and grasslands.

**TABLE 1:** Summary of MUA analysis of six troops with the original observation period (from Start date to End date)

Troop ID		T1	T2	T3	T4	T5	T6
Site		Shikoku	Honshu	Shikoku	Honshu	Honshu	Honshu
Troop size (individuals)		U	52	U	73	57	15
Altitude^a^ (m)		465.4	136.2	617.7	186.7	75.2	218.7
Temperature^b^ (°C)		16.1	19.2	9.7	9.7	14.7	12.5
Vegetation		P	D	P	P	D	D
Start date^c^		06/02/15	07/02/16	27/11/13	31/01/15	29/08/15	01/09/16
End date^c^		05/10/15	24/10/16	12/06/14	08/05/15	26/04/16	12/06/17
Observation period^d^ (days)		242	260	172	98	239	283
MCP^e^ (km^2^)		18.5	13.5	14.2	98.0	4.4	9.8
*y*_MUA_ (km^2^)	Cum	22.3	18.0	1516	9670	4.5	10.2
	Avg	21.5	14.6	18.3	282	3.1	8.4
*y*_365_ (km^2^)	Cum	19.1	14.9	29.2	370	4.5	10.1
	Avg	18.9	13.5	15.1	200	3.0	7.6
*k* (days)	Cum	61.8	77.7	18575	9174	4.6	5.9
	Avg	50.8	30.4	79.6	150	8.4	41.1
*b*	Cum	0.42	0.44	0.94	1.20	0.11	0.11
	Avg	0.43	0.34	0.55	0.80	0.23	0.38

^a^ The mean altitude was obtained from the Conservation GIS consortium Japan (http://cgisj.jp/) based on the digital elevation map of the Geospatial Information Authority of Japan (http://www.gsi.go.jp/ENGLISH/index.html).

^b^ The mean temperature during the observation period was obtained from the Japan Meteorological Agency (http://www.jma.go.jp/jma/menu/menureport.html).

^c^ Dates are expressed as dd/mm/yy.

^d^ Observation period could be shorter than the number of days of observation, because days with <5 locations sampled were removed from the analysis.

^e^ 100%‐MCP area calculated from the full observation period. U: unknown; P: plantation (dominated by *Cryptomeria japonica* or *Chamaecyparis obtusa*), D: secondary deciduous broadleaf forest (dominated by *Quercus serrata*); Cum: cumulative method, Avg: averaged method.

In each troop, location data were collected from one adult female with a GPS data‐logging collar (Tellus 1C light, Followit, Lindesberg, Sweden; GLT‐02, Circuit Design, Nagano, Japan) because adult females are unlikely to leave their troops (Izumiyama et al., 2003). The collar weight was 210 g (Tellus 1C light) or 250 g (GLT‐02), which were less than 10% of the animal's weight, following the guideline for field research of non‐human primates (Primate Research Institute, Kyoto University, 2019). To fit the animal with a collar, monkeys were captured by corral trap (T1), foothold trap (T3), or tranquilizer guns (T2, T4–T6), following the guidelines for the procedure of obtaining mammal specimens as approved by the Mammal Society of Japan (The Committee of Reviewing Taxon Names and Specimen Collections, Mammal Society of Japan, 2009). The GPS data‐logger was programed to log the location daily every hour between 6:00 and 18:00 (13 location data per day). Some location data were not obtained due to poor radio reception. As is often the case with location data with intervals of ≤4 h (e.g., Lesilau et al., 2021; Moland et al., 2011; Pascoe et al., 2018), the observation periods of these troops were shorter than 1 year due to the limited battery life.

### 2.2 Estimation of maximum utilization area

The MUA was estimated by two distinct methods (cumulative vs. averaged methods) and compared. In the cumulative method, the relationship between utilization area and days of observation (duration) is simply plotted from the start day to the end day (i.e., area–duration curve). In the averaged method, utilization areas were averaged over all possible combinations of the duration by obtaining subsamples from the original dataset to reduce daily variation in utilization area. For example, there is a dataset with *n* days of observation period. The utilization area for a single day in this dataset can be obtained from *n* ways (i.e., *n* subsamples). The utilization area for two days in this dataset can be obtained from *n* – 1 combinations of two consecutive days (i.e., Days 1–2, Days 2–3, …, Days [*n* − 2]–[*n* − 1], Days [*n* − 1]–[*n*]). Similarly, the utilization area of *i* consecutive days in this dataset can be obtained from *n* – *i* + 1 combinations of the duration (i.e., *n* – *i* + 1 subsamples). To save computation time, the durations (*i*) to calculate utilization areas were increased with intervals of one day when *i* ≤ 60, with intervals of 5 days when 60 < *i* ≤ 90, with intervals of 10 days when *i* > 90 in both methods. The area–duration curve was represented by a Michaelis–Menten equation:

(1) $$y=\frac{\;y_{\mathrm{MUA}}\;x}{k+x}$$

where *y* is the cumulative or averaged utilization area (km^2^), *x* is duration (days), *y*_MUA_ is the asymptotic value of utilization area when *x* → ∞, and *k* is a half‐saturation constant (days). Because many animals have their home ranges on a timescale of one year (e.g., Itani & Tokuda, 1954; Walton et al., 2001), we here introduce an index *y*_365_, the utilization area with duration of timescale (*x* = 365), to check if the curve reaches an asymptote with 1 year. Coefficients (*y*_MUA_ and *k*) were estimated with the "drc" package in R version 3.6.1 (R Core Team, 2019; Ritz & Streibig, 2005).

To obtain plots of utilization area against duration, the utilization area was calculated by 100% MCP using the package "adehabitat" in r (Calenge, 2006). Because the package requires at least five locations to calculate a utilization area, days with data less than five locations were excluded from the analysis.

### 2.3 Manipulation of observation period

Animals often have different utilization sites depending on time of year (Hanya et al., 2006; Kozakai et al., 2017; Prange et al., 2004; Rivrud et al., 2010). To investigate effects of start and end dates, season, and observation period on the estimated MUA (*y*_MUA_), we experimentally reduced the observation period by removing days from the end date in descending order of date or from the start date in ascending order. With manipulated data, we estimated *y*_MUA_ in cumulative and averaged methods using Equation (1).

The relationship between the shape of the area–duration curve and *y*_MUA_ was characterized using the package "drc" by a power function:

(2) $$y=ax^{b}$$

where *y* is the cumulative or averaged utilization area (km^2^), *x* is duration (days), and *a* and *b* are positive constants. Equation (2) is an acceleratingly increasing function when *b* > 1, linear function when *b* = 1, and deceleratingly increasing function when *b* < 1. Thus, the exponent *b* expresses a measure of curvature and we experimentally tested the relationship between *y*_MUA_ and *b* using manipulated datasets that include a variety of seasons and observation periods.

## 3 RESULTS

### 3.1 MUA of full observation period

**FIGURE 1:**
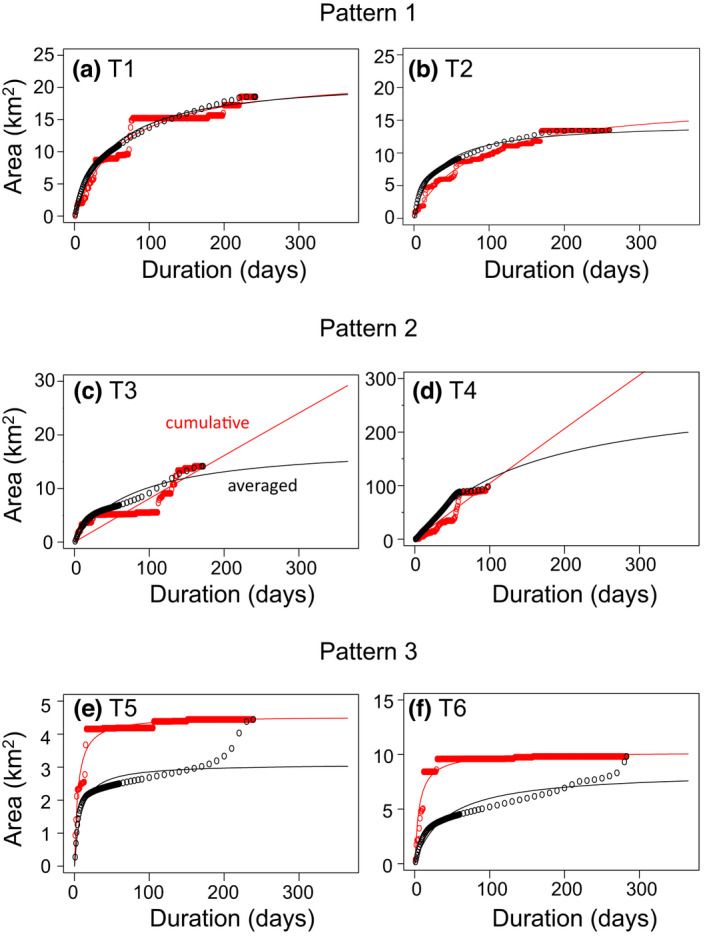
Utilization area versus duration of six troops (a–f) calculated based on the cumulative method (red plots) and averaged method (black plots). Red and black lines represent the area–duration curves using the Michaelis–Menten equation (Equation 1)

The full observation periods of the six monkey troops ranged from 98 to 283 days (Table 1). Areas of the full observation periods calculated by the 100% MCP ranged from 4.4 to 98 km^2^. The estimated MUA (i.e., *y*_MUA_) based on the area–duration curve in the cumulative and averaged methods exhibited three patterns (Figure 1, Table 1). Pattern 1: there was little difference between *y*_MUA_ and *y*_365_ for both methods (Troops T1 and T2). Pattern 2: half‐saturation constant (*k*) was much greater than 1 year (365 days) in the cumulative method, resulting in large values of *y*_MUA_ compared to *y*_365_ (*y*_MUA_/*y*_365_ > 2, T3 and T4). Pattern 3: *y*_MUA_ estimated with the averaged method was smaller than the area calculated by the 100% MCP (T5 and T6). In Pattern 3, the area–duration curve in the averaged method showed an accelerating increase at the end of the observation period. The exponent *b* took values close to or >1 when *y*_MUA_ took extremely large values, otherwise it was considerably smaller than 1.

### 3.2 MUA of manipulated observation period

**FIGURE 2:**
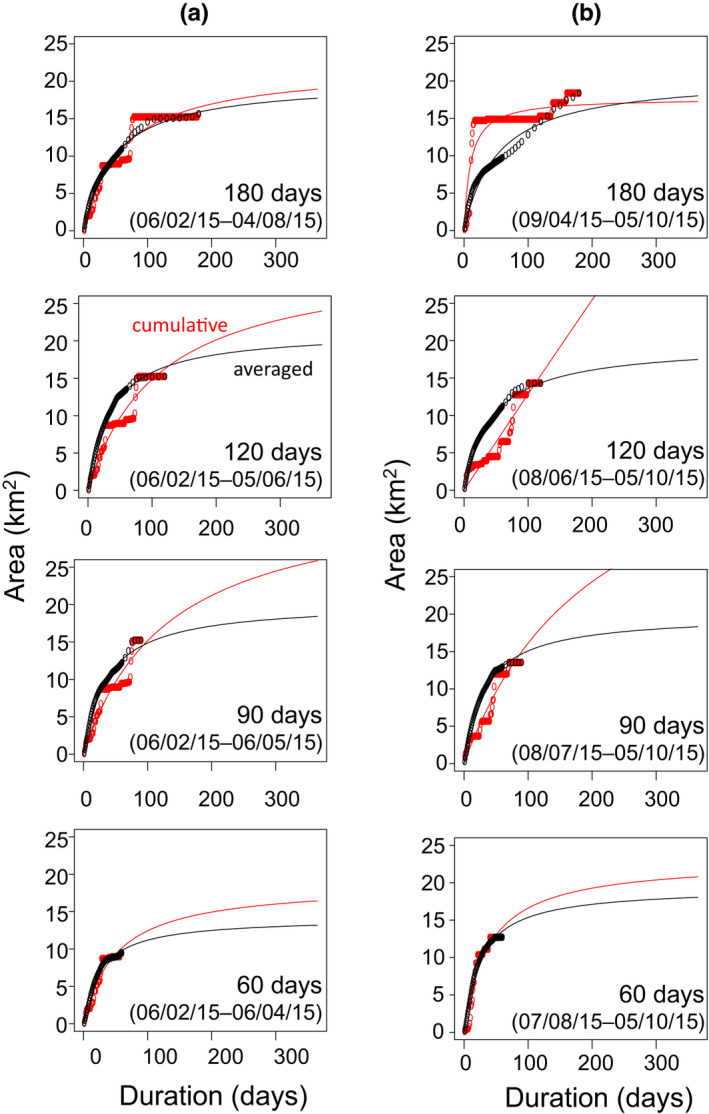
Area–duration plots and curves (Equation 1) with manipulated observation period in the case of T1 (Patten 1), calculated based on the cumulative method (red) and averaged method (black). The observation period was manipulated by (a) removing days from the end date in descending order and (b) removing days from the start date in ascending order (180, 120, 90, and 60 days). Dates are expressed as dd/mm/yy

With changes in the observation period, *y*_MUA_ calculated with the averaged method showed small variation in Pattern 1 (Figure 2, Table 2). In contrast, *y*_MUA_ calculated with the cumulative method was sensitive to changes in the observation period. With the cumulative method, reductions of days in ascending order sometimes resulted in large values of *y*_MUA_ as shown in Pattern 2 (120 days; Figure 2b).

**TABLE 2:** Summary of MUA analysis of troops 1 and 5 (T1 and T5) with manipulated observation periods (the original observation period was reduced to 180, 120, 90, and 60 days in descending or ascending order)

		T1				T5			
		*y*_MUA_		*k*		*y*_MUA_		*k*	
		Cum	Avg	Cum	Avg	Cum	Avg	Cum	Avg
180 d	Desc	22.0	20.0	59.9	47.1	4.5	3.1	4.6	7.1
	Asc	17.7	20.6	10.0	53.2	2.5	2.6	2.5	5.5
120 d	Desc	31.2	21.4	110.5	53.2	4.5	3.1	4.5	5.7
	Asc	1324	19.6	10209	35.4	2.8	2.7	8.0	5.8
90 d	Desc	35.3	20.2	133	35.4	4.6	3.4	4.7	6.6
	Asc	49.7	19.9	209	43.7	2.7	2.6	4.4	5.4
60 d	Desc	18.7	14.1	50.5	43.7	4.8	4.6	5.6	10.6
	Asc	23.0	19.3	38.4	36.3	3.0	2.5	13.5	4.9

Note

Cum and Avg are cumulative and averaged methods, respectively. Desc and Asc are reductions of days in descending and ascending orders, respectively.

**FIGURE 3:**
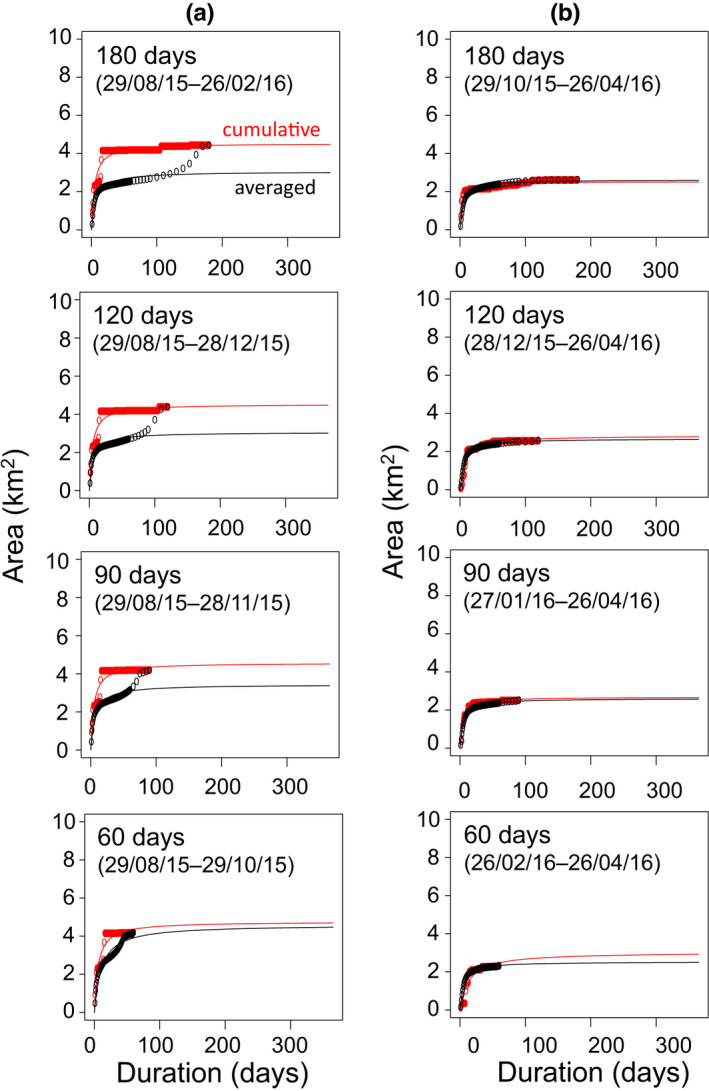
Area–duration plots and curves (Equation 1) with manipulated observation period in the case of T5 (Pattern 3), calculated based on the cumulative method (red) and averaged method (black). The observation period was reduced by (a) removing days from the end date in descending order and (b) removing days from the start date in ascending order (180, 120, 90, and 60 days). Dates are expressed as dd/mm/yy

When the observation period was reduced in descending order for the troops exhibiting Pattern 3, the area–duration curves did not qualitatively differ from the curve with the original (non‐manipulated) observation period in terms of *y*_MUA_ in the averaged method being smaller than the 100%‐MCP area, small *k* in the cumulative method, and an accelerating increase at the end of observation period in the averaged method (180, 120, and 90 days; Figure 3a, Table 2). When the observation period was reduced in ascending order, the area versus duration plots showed typical saturation curves with both methods (Figure 3b). Reductions of days in ascending order resulted in relatively small values of *y*_MUA_, compared to *y*_MUA_ with the original observation period (Tables 1 and 2).

**FIGURE 4:**
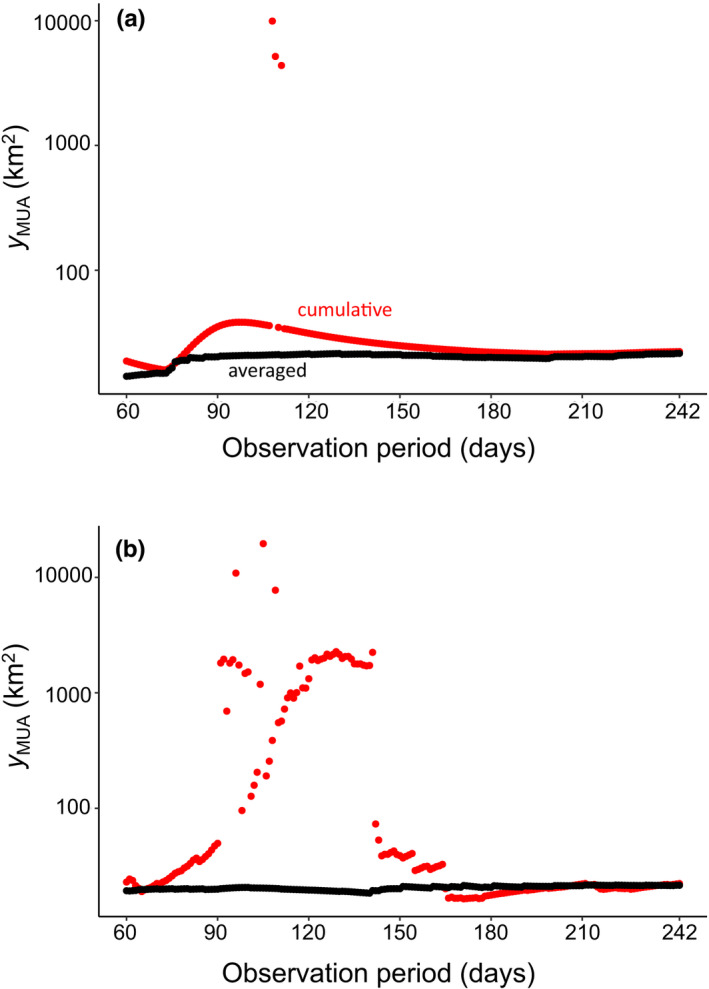
Relationships between estimated MUA and observation period in the cumulative (red plots) and averaged (black plots) methods in the case of T1 (Pattern 1). The observation period was reduced (a) in descending order and (b) in ascending order. Note that *y*‐axes are expressed on a log‐scale

Effects of reducing the observation period on *y*_MUA_ were shown in Figure 4. The values of *y*_MUA_ in the averaged method were robust against changes in the observation period (16–22 km^2^), while *y*_MUA_ in the cumulative method widely varied (16–19,600 km^2^). In the cumulative method, reductions of days in ascending order resulted in larger variation of *y*_MUA_ than reductions in descending order.

**FIGURE 5:**
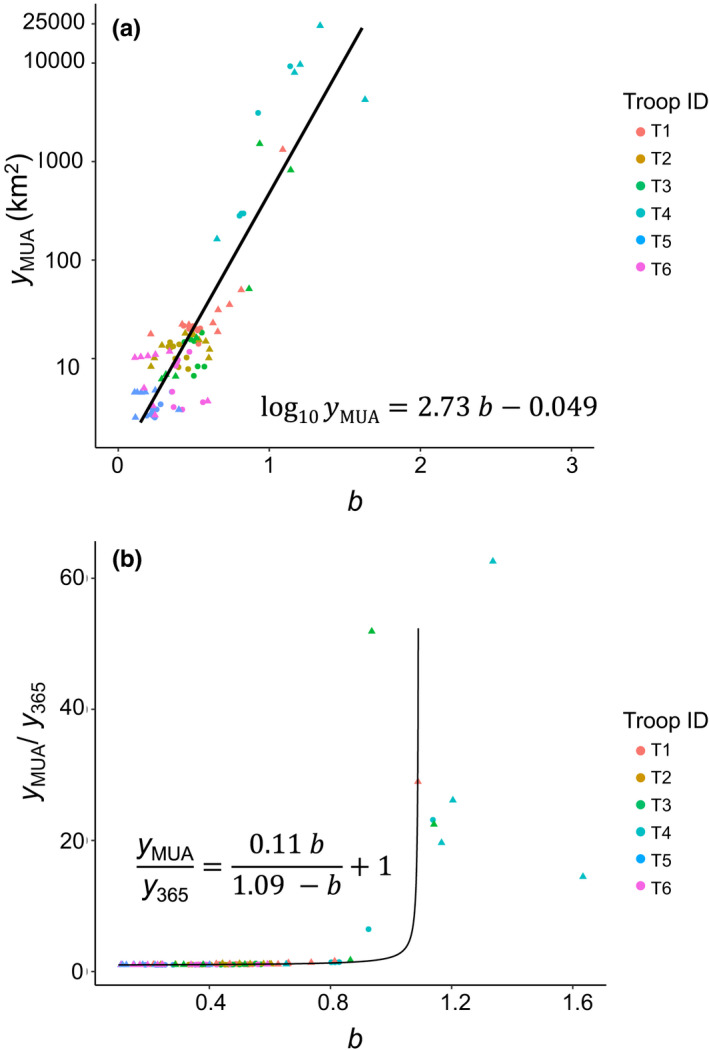
Estimated MUA in relation to the exponent *b*, using non‐manipulated and manipulated data of six troops, calculated based on both cumulative (triangles) and averaged (circles) methods. The manipulated data included reductions of days in both descending and ascending orders. Manipulated observation periods, depending on their original periods, are as follows. T1, T5, and T6: 180, 120, 90, and 60 days; T2 and T3: 120, 90, and 60 days; T4: 90 and 60 days. (a) Relationship between *y*_MUA_ on log‐scale and *b*, along with a linear regression. (b) Relationship between *y*_MUA_/*y*_365_ and *b*. The regression equation was obtained based on a Michaelis–Menten equation ($y=\frac{\mathrm{cx}}{d+x}$ where *y* = *b*, $x=\frac{y_{\mathrm{MUA}}}{y_{365}}‐1$, *c* = 1.09 and *d* = 0.11)

Plots of estimated MUA (*y*_MUA_ on log‐scale) against exponent *b* showed a log‐linear relationship (Figure 5a). The relationship showed, for example, that *b* = 0.38 when *y*_MUA_ = 10 and *b* = 0.75 when *y*_MUA_ = 100. When *b* = 1, *y*_MUA_ = 479 km^2^. There was a non‐linear relationship between *y*_MUA_/*y*_365_ and *b* (Figure 5b). Using the regression equation obtained based on a Michaelis–Menten equation, *b* was calculated at 0.98 when *y*_MUA_/*y*_365_ = 2 and at 0.90 when *y*_MUA_/*y*_365_ = 1.5.

## 4 DISCUSSION

We here estimated the MUA, which is important for applied ecology such as wildlife conservation and management, based on our new method (i.e., averaged method) as well as the conventional method (cumulative method). Our results showed that the averaged method, compared to the cumulative method, provided reliable estimates for MUA. The estimated MUA (*y*_MUA_) had three patterns. Troops exhibiting Pattern 1 (T1 and T2) were sufficiently sampled to estimate MUA. Pattern 2, in which the area–duration curve in the cumulative method did not reach an asymptote with a duration of 1 year, was produced for troops exhibiting Pattern 1 when the observation period was reduced. According to the concept that estimates of utilization area should reach an asymptote with an adequate sample size (Laver & Kelly, 2008), this result indicates that the observation period was not sufficiently long enough to accurately estimate MUA for troops exhibiting Pattern 2 (T3 and T4). For troops exhibiting Pattern 3 (T5 and T6) in which MUA in the averaged method had a tendency to be an underestimate, their distributions of monthly utilization sites considerably overlapped and utilization sites at the beginning of observation were relatively large (see Figure S1). The peak of the monthly utilization area differed among Patterns 1–3: in between the observation period for Pattern 1, at the end of the period for Pattern 2 (except for T4 with a limited period), and at the beginning of the period for Pattern 3 (see Figure S2). Hanya et al. (2006) reported that many Japanese monkeys utilize relatively large sites in summer and autumn (i.e., June–November), but some monkeys do not. In this study, the peak of the monthly utilization area depended on the troops. For example, the peak for T1 was spring and the peak for T2 was summer (see Figure S2). These results suggest that Patterns 1 and 2 hinge on the location of the peak, rather than the season included in the dataset, when the observation period is sufficiently long. When we manipulated the T1 dataset such that it started on 1 April and ended on 31 July, the manipulated dataset exhibited Pattern 3 (see Figure S3).

With the cumulative method, *y*_MUA_ was shown to be more sensitive to reductions of days in ascending order than reductions in descending order. This result suggests that MUA estimates are likely to depend on the start date. To check if an area–duration curve reaches an asymptote with a given observation period and start date, the exponent *b* (Equation (2)) is a useful index. The exponent took a value close to 1 when *y*_MUA_/*y*_365_ = 2. In this case, area increases with duration in a linear manner, indicating that the utilization area does not approach an asymptote with the observation period and start date. Area–duration curves with *b* < 0.9, corresponding to *y*_MUA_/*y*_365_ < 1.5, may be considered as those that approach an asymptote with the observation period and start date. In this study, *y*_365_ was used as an index for duration, because Japanese monkeys are known to have home ranges on a timescale of 1 year (Itani & Tokuda, 1954). Home ranges on 1‐year timescale are also known for other animals such as wolves (Walton et al., 2001), wild turkeys (Hall et al., 2006), coyotes (Gehrt et al., 2009), giraffes (Flanagan et al., 2016), and takins (Yan et al., 2017). Home ranges on shorter timescales were reported in Norway rats (2 months; Oyedele et al., 2015) and in feral cats (8–46 days; Leo et al., 2016). In such animals, an alternative index for duration may be applied (e.g., 60 days, *y*_60_).

In contrast to the cumulative method, MUA calculated with the averaged method were robust against changes in observation period or start or end date. However, analysis of monthly utilization sites showed that *y*_MUA_ estimates calculated with the averaged method were likely an underestimate when the observation started in an actively foraging season. In such a case, application of the 100% MCP or cumulative method would result in a more precise estimate for MUA, rather than application of the averaged method. In general, daily foraging areas of animals are variable depending on season and weather conditions (Rivrud et al., 2010; van Beest et al., 2011). The utilization area of a single day, corresponding to the area on the start date in the manipulation of reducing days in ascending order, varied over two orders of magnitude, for example, for T1 (range 0.01–1.62 km^2^, average 0.27 km^2^; see Figure S4). In the case of T1, for the cumulative method, the estimated utilization area on the first day can stochastically take a value ranging from 0.01 to 1.62 depending on the start date of observation. With the averaged method, the estimate is always 0.27 irrespective of the start date. Thus, MUA estimates in the cumulative method varied greatly depending on the observation period and start date because the method is subject to the effect of temporal stochasticity in animal movements. The averaged method reduced the effect of temporal stochasticity by averaging the areas calculated from all possible combinations, resulting in considerably stable estimates for MUA.

In this paper, we used Japanese monkeys as our model species. The averaged method was tested for the estimation of MUA in other mammals. MUA estimates in the averaged method were more stable than those in the cumulative method when both methods were applied to the red fox (*Vulpes vulpes*) with limited observation periods (22–105 days; see Table S1, Figure S5). In the bobcat (*Lynx rufus*) with sufficient observation periods (>200 days), MUA estimates with the averaged method were more stable against manipulations of reducing the observation period than those with the cumulative method (Figure S7), although both methods resulted in similar MUA estimates when the full observation period was used (Table S1, Figure S6). In addition, we applied the averaged method to simulated data. With simulated data created by a biased correlated random walk, it was shown that the averaged method more stably provided reliable estimates for MUA than the cumulative method (see Figure S8). Application of the averaged method improves MUA estimates in cases where the data were collected within a limited observation period and where daily or seasonal foraging activity of the study animal is highly variable.

## AUTHOR CONTRIBUTIONS

**Kana Terayama:** Conceptualization (equal); Data curation (lead); Formal analysis (lead); Methodology (equal); Writing – original draft (equal); Writing – review & editing (equal). **Hiroshi Ebihara:** Investigation (lead); Resources (equal); Writing – review & editing (supporting). **Hironori Seino:** Investigation (supporting); Resources (equal); Supervision (supporting). **Motomi Genkai‐Kato:** Conceptualization (equal); Methodology (equal); Supervision (lead); Writing – original draft (equal); Writing – review & editing (equal).

## Supporting information

Supinfo

## Data Availability

The location data and R codes used in this study are available from the Dryad data repository (https://doi.org/10.5061/dryad.zs7h44jc8).
