# Supplementary material for: Estimation of the maximum utilization area including home range and peripheral sites
Source: Ecol Evol. 2022 May 7;12(5):e8893. doi: 10.1002/ece3.8893 (PMC9077728; doi:10.1002/ece3.8893)
Supplement: Supplementary file 1 — Supinfo [file ECE3-12-e8893-s001.docx]

**Figure S1.** Distribution map of monthly utilization sites of six troops (a–f). Months with < 20 days at the beginning or end of observation period were removed from the distribution map.

**Figure S2.** Monthly utilization area of the six troops (T1–F6). Months with < 20 days at the beginning or end of observation period were not shown.

**Figure S3.** Area–duration plots and curves (Equation (1)) with manipulated observation period in the case of T1 (Patten 1), calculated based on the cumulative method (red) and averaged method (black). The observation starts on 1 April and ends on 31 July. The estimated MUA (*y*_MUA_) is 18.8 km^2^ (cumulative method) and 17.0 km^2^ (averaged method). The estimated MUA with the averaged method is smaller than the area calculated by the 100% MCP (18.5 km^2^). The area–duration curve in the averaged method showed an accelerating increase at the end of the observation period as shown in Pattern 3.

**Figure S4.** Utilization area of one day in six troops (a–f). Area ranged (a) 0.01–1.62 km^2^ (mean ± SD: 0.27 ± 0.27 km^2^), (b) 0.01–1.28 km^2^ (0.32 ± 0.22 km^2^), (c) 0.01–1.02 km^2^ (0.18 ± 0.16 km^2^), (d) 0.02–2.06 km^2^ (0.49 ± 0.40 km^2^), (e) 0.01–0.94 km^2^ (0.27 ± 0.20 km^2^), (f) 0.02–0.83 km^2^ (0.17 ± 0.13 km^2^).

**Table S1.** Summary of MUA analysis of the bobcat (*Lynx rufus*: L1 and L2) and the red fox (*Vulpes vulpes*: V1–6) with the original observation period.

| ID |  | L1 | L2 | V1 | V2 | V3 | V4 | V5 | V6 |
| --- | --- | --- | --- | --- | --- | --- | --- | --- | --- |
| Observation period (days) |  | 259 | 210 | 105 | 103 | 90 | 80 | 80 | 22 |
| MCP (km^2^) |  | 35.2 | 59.0 | 132.1 | 126.2 | 325.1 | 17.7 | 42.0 | 9.9 |
| *y*_MUA_ (km^2^) | Cum | 33.2 | 69.7 | 154.7 | 38008.2 | 516.6 | 19.2 | 46.6 | 12.8 |
|  | Avg | 30.9 | 59.3 | 197.5 | 178.9 | 510.6 | 15.3 | 38.5 | 10.5 |
| *y*_365_ (km^2^) | Cum | 32.7 | 64.2 | 150.5 | 495.7 | 468.5 | 18.5 | 45.6 | 12.6 |
|  | Avg | 29.2 | 56.0 | 161.9 | 162.9 | 445.5 | 15.2 | 37.9 | 10.4 |
| *k* (days) | Cum | 5.5 | 31.2 | 10.2 | 27619.3 | 37.4 | 15.1 | 7.9 | 5.9 |
|  | Avg | 21.7 | 21.5 | 80.3 | 35.9 | 53.3 | 3.0 | 6.1 | 1.6 |
| *b* | Cum | 0.13 | 0.33 | 0.26 | 1.23 | 0.53 | 0.37 | 0.27 | 0.43 |
|  | Avg | 0.31 | 0.33 | 0.61 | 0.50 | 0.61 | 0.19 | 0.26 | 0.21 |

**Figure S5.** Utilization area versus duration of six individuals (V1–6) of the red fox, *Vulpes vulpes*, calculated based on the cumulative method (red plots) and averaged method (black plots). Red and black lines represent the area–duration curves using the Michaelis–Menten equation (Equation (1)). The data used in this study were published in the Movebank Data Repository (Cater & David, 2020; Cater et al., 2019), where V1–6 correspond to "Kev", "Bea", "Jim", "Ern", "Ros" and "Kim", respectively.

**Figure S6.** Utilization area versus duration of two individuals (L1 and L2) of the bobcat, *Lynx rufus*, calculated based on the cumulative method (red plots) and averaged method (black plots). Red and black lines represent the area–duration curves using the Michaelis–Menten equation (Equation (1)). The data used in this study are available on Movebank (movebank.org, study name "Carnivore movements near Black Rock Forest New York", study ID 1088836380), where L1 and L2 correspond "LYRU_0001" and "LYRU_0002", respectively.

**Figure S7.** Relationships between estimated MUA (*y*_MUA_) and observation period in the cumulative (red plots) and averaged (black plots) methods in the case of L1 (*Lynx rufer*). The observation period was reduced (a) in descending order and (b) in ascending order.

**Figure S8.** Percentage error of MUA estimates in the cumulative and averaged methods from the true MUA (MUA_true_) when the duration was reduced from 1000 days to 365, 180, 120, 90 and 60 days, using simulated data created by a biased correlated random walk (BCRW; Long et al., 2014). Location data were generated with BCRW parameters based on Gilbertson et al. (2020): *h* = 34, *ρ* = 0.8, *b* = 0.01, and *c* = 0.3. We assumed that there were 13 location data per day as in the text and that MUA_true_ was the 100%-MCP area for the duration of 1000 days (corresponding to about three years). We ran 1000 simulations. Percentage of simulations where MUA estimates exceeded (a) 2 × MUA_true_, and (b) 10 × MUA_true_. We performed all simulations in R, version 3.6.1 (R Core Team, 2019). BCRW R code was adapted from Gilbertson et al. (2020).

**References**

Cater, A., & David, A. R. 2020 Data from: Toward reliable population density estimates of partially marked populations using spatially explicit mark-resight methods. Movebank Data Repository. https://doi.org/10.5441/001/1.72hh609t

Cater, A., Potts, J. M., & Roshier, D. A. 2019 Toward reliable population density estimates of partially marked populations using spatially explicit mark-resight methods. *Ecology and Evolution*, 9, 2131-2141. https://doi.org/10.1002/ece3.4907

Gilbertson M. L. J., White L. A., & Craft M. E. 2020 Trade-offs with telemetry-derived contact networks for infectious disease studies in wildlife. *Methods in Ecology and Evolution*, 12, 76-87. https://doi.org/10.1111/2041-210X.13355

Long J. A., Nelson T. A., Webb, S. L., & Gee K. L. 2014 A critical examination of indices of dynamic interaction for wildlife telemetry studies. Journal of Animal Ecology, 83, 1216-1233. https://doi.org/10.1111/1365-2656.12198

R Core Team. (2019). *R: a language and an environment for statistical computing* (Version 3.6.1). R Foundation for Statistical Computing. Retrieved from https://www.r-project.org
